# Evidence That Ca^2+^ within the Microdomain of the L-Type Voltage Gated Ca^2+^ Channel Activates ERK in MIN6 Cells in Response to Glucagon-Like Peptide-1

**DOI:** 10.1371/journal.pone.0033004

**Published:** 2012-03-07

**Authors:** Joanne Selway, Roberto Rigatti, Nina Storey, Jing Lu, Gary B. Willars, Terence P. Herbert

**Affiliations:** Department of Cell Physiology and Pharmacology, Henry Wellcome Building, University of Leicester, Leicester, United Kingdom; University of Houston, United States of America

## Abstract

Glucagon like peptide-1 (GLP-1) is released from intestinal L-cells in response to nutrient ingestion and acts upon pancreatic β-cells potentiating glucose-stimulated insulin secretion and stimulating β-cell proliferation, differentiation, survival and gene transcription. These effects are mediated through the activation of multiple signal transduction pathways including the extracellular regulated kinase (ERK) pathway. We have previously reported that GLP-1 activates ERK through a mechanism dependent upon the influx of extracellular Ca^2+^ through L-type voltage gated Ca^2+^ channels (VGCC). However, the mechanism by which L-type VGCCs couple to the ERK signalling pathway in pancreatic β-cells is poorly understood. In this report, we characterise the relationship between L-type VGCC mediated changes in intracellular Ca^2+^ concentration ([Ca^2+^]_i_) and the activation of ERK, and demonstrate that the sustained activation of ERK (up to 30 min) in response to GLP-1 requires the continual activation of the L-type VGCC yet does not require a sustained increase in global [Ca^2+^]_i_ or Ca^2+^ efflux from the endoplasmic reticulum. Moreover, sustained elevation of [Ca^2+^]_i_ induced by ionomycin is insufficient to stimulate the prolonged activation of ERK. Using the cell permeant Ca^2+^ chelators, EGTA-AM and BAPTA-AM, to determine the spatial dynamics of L-type VGCC-dependent Ca^2+^ signalling to ERK, we provide evidence that a sustained increase in Ca^2+^ within the microdomain of the L-type VGCC is sufficient for signalling to ERK and that this plays an important role in GLP-1- stimulated ERK activation.

## Introduction

GLP-1 is a hormone secreted from intestinal L-cells in response to nutrients (for reviews see [1], [2]). The primary site of GLP-1 action is the pancreatic β-cell where it stimulates proliferation, differentiation, insulin gene transcription, and the potentiation of glucose-dependent insulin secretion. These effects are mediated through the activation of the GLP-1 receptor (GLP-1R), a G_αs_-protein coupled receptor of the secretin/glucagon type B family. GLP-1R agonists stimulate an increase in the production of cAMP, which potentiates glucose-stimulated rises in intracellular free [Ca^2+^] ([Ca^2+^]_i_), and thus insulin secretion through a number of mechanisms including the inactivation of K^+^_ATP_ channels. GLP-1 also potentiates glucose-stimulated ERK activation [3]–[7], and importantly the activation of ERK has been shown to play a positive role in the stimulation of pancreatic β-cell proliferation, differentiation, survival and insulin gene transcription [7]–[9]. Furthermore, in the pancreatic β-cell line MIN6 (mouse insulinoma 6), glucose-induced ERK1/2 activation enhances insulin secretion via the phosphorylation of synapsin I [10].

We have previously reported that GLP-1-stimulated ERK activation in β-cells is dependent on the influx of Ca^2+^ through the L-type VGCC, as nifedipine, an L-type VGCC blocker, effectively inhibits GLP-1-induced ERK activation [4]. Moreover, other agents that activate L-type VGCCs, such as glucose, depolarising concentrations of K^+^ or Bay-K 8644, an L-type VGCC agonist, all lead to the activation of ERK via a nifedipine-sensitive pathway [3]–[6], [11]. However, the mechanism by which GLP-1 signals to ERK via the L-type VGCC is unknown.

In this report, we provide evidence that, in the pancreatic β-cell line Mouse insulinoma-6 cells (MIN6 cells) [12], GLP-1 stimulates ERK phosphorylation through a mechanism requiring the sustained activation of L-type VGCCs but which does not require a sustained global increase in [Ca^2+^]_i_. Instead, we show that a sustained increase in [Ca^2+^] within the microdomain of the L-type VGCC is sufficient for L-type VGCC signalling to ERK activation and plays an important role in the sustained phase of ERK activation in response to GLP-1.

## Results

### GLP-1 stimulated ERK activation requires L-type VGCC activation

**Figure 1:**
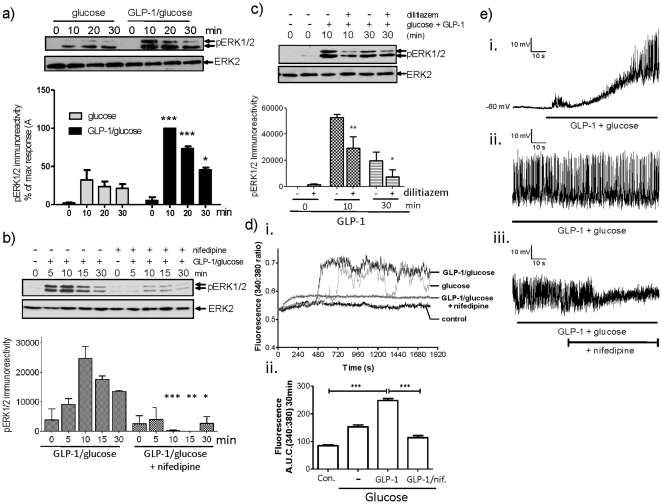
GLP-1-stimulated ERK activation requires L-type VGCC activation. a) MIN6 cells were preincubated for 1 h in KRB supplemented with 1 mM glucose. Cells were then incubated in 16.7 mM glucose in the absence or presence of 10 nM GLP-1. Proteins were resolved by SDS-PAGE and Western blotted using anti-phospho-ERK1/2 (pERK) or anti-ERK2 (ERK2) antibodies. A representative blot is shown above densitometric analysis of the results showing mean +S.E.M. (n = 5). All statistical comparisons were by one-way ANOVA with Bonferroni's multiple comparison test compared to glucose alone at each time point; *, *P*<0.05; **, *P*<0.01; ***, *P*<0.001. b) MIN6 cells were preincubated for 1 h in KRB supplemented with 1 mM glucose. Cells were stimulated with 10 nM GLP-1 plus 16.7 mM glucose in the presence or absence of 10 µM nifedipine for the times indicated. Proteins were resolved by SDS-PAGE and Western blotted using anti-phospho-ERK1/2 and anti-ERK2 antibodies. A representative blot is shown above densitometric analysis of the results showing mean +S.E.M. (n = 3). All statistical comparisons were by one-way ANOVA with Bonferroni's multiple comparison test compared to GLP-1 plus glucose at each time point; *, *P*<0.05; **, *P*<0.01; ***, *P*<0.001. c) MIN6 cells were preincubated for 1 h in KRB supplemented with 1 mM glucose. Cells were stimulated with 10 nM GLP-1 plus 16.7 mM glucose in the presence or absence of 50 µM dilitiazem for the times indicated. Cell lysates were analysed by SDS-PAGE and Western blotting using anti-phospho-ERK1/2 or anti-ERK2 antibodies. A representative blot is shown above densitometric analysis of the results ± S.E.M. All statistical comparisons were by one-way ANOVA with Bonferroni's multiple comparison test compared to glucose plus GLP1; *, *P*<0.05; **, *P*<0.01; (n = 3). d) MIN-6 cells were loaded with fura-2-AM and [Ca^2+^]_i_ levels measured using epifluorescence microscopy. i) Representative traces from single cells incubated with 1 mM glucose (control), 16.7 mM glucose (glucose), 10 nM GLP-1 plus 16.7 mM glucose (GLP-1/glucose) or 10 nM GLP-1 plus 16.7 mM glucose in the presence of 10 µM nifedipine (GLP-1/glucose + nifedipine). ii) Area under the curve (A.U.C.) across the 30 min stimulation showing mean +S.E.M. (n>30). Statistical comparisons were by one-way ANOVA with Dunnett's range test compared to GLP-1 plus glucose; ***, *P*<0.001. e) Membrane potential recordings from MIN6 cells recorded in the perforated-patch, current-clamp mode. i) The effect of addition of 10 nM GLP-1 plus 16.7 mM glucose on membrane potential. ii) A membrane potential recording after 5.5 min in the continuous presence of 10 nM GLP-1 and 16.7 mM glucose. iii) After 10 min in the presence of 10 nM GLP-1 and 16.7 mM glucose, the effect on excitability of bath application of nifedipine (10 µM) was recorded.

GLP-1 signalling to ERK was characterised in the pancreatic β-cell line MIN6 [12]. Cells were incubated with 16.7 mM glucose in the presence and absence of 10 nM GLP-1 for up to 30 min and changes in the phosphorylation status of ERK were determined as an index of ERK activation. As previously reported [4], glucose led to an increase in ERK activation, which was potentiated by GLP-1 (Figure 1a). To confirm the dependency of GLP-1-stimulated ERK phosphorylation on L-type VGCC channel activation, MIN6 cells were treated with glucose plus GLP-1 for up to 30 min in the presence or absence of the L-type VGCC blocker, nifedipine (Figure 1b). GLP-1 plus glucose treatment of MIN6 cells resulted in the rapid phosphorylation of ERK that remained elevated for the duration of the experiment. These increases in ERK phosphorylation were inhibited by nifedipine at all time points tested. Similar results were obtained using another L-type VGCC blocker dilitiazem (Figure 1c). These results confirm that in MIN6 cells, GLP-1-stimulated ERK phosphorylation in the presence of an elevated concentration of glucose requires L-type VGCC activation. In order to investigate the temporal correlation of GLP-1 plus glucose-stimulated L-type VGCC-dependent ERK activation with increases in [Ca^2+^]_i_, changes in [Ca^2+^]_i_ were assessed by single-cell epifluorescence microscopy in response to glucose, GLP-1 plus glucose or GLP-1 plus glucose in the presence of nifedipine. Application of 16.7 mM glucose alone caused a rise in [Ca^2+^]_i_ initiating approximately 5 min after application which, like ERK activation, was potentiated by 10 nM GLP-1 (Figure 1d). The co-application of nifedipine blocked GLP-1 plus glucose stimulated increases in [Ca^2+^]_i_ (Figure 1d). Using an electrophysiological approach, we also show that GLP-1 plus glucose application caused membrane depolarisation (to −21±2 mV from −62.5±2.2 mV; n = 17.cells) and action potential firing (figure 1e i and ii). Application of nifedipine did not cause membrane repolarization but did cause a loss of action potential firing (Figure 1e iii). This is consistent with recordings form human pancreatic β-cells in which a Ca^2+^ channel blocker, isradipine, caused action potential failure [13]. These data provide evidence that the activation of L-type VGCCs play an important role in the membrane excitability and that increases in [Ca^2+^]_i_ coincide with increased ERK phosphorylation.

### The sustained activation of L-type VGCCs is required for the sustained activation of ERK

**Figure 2:**
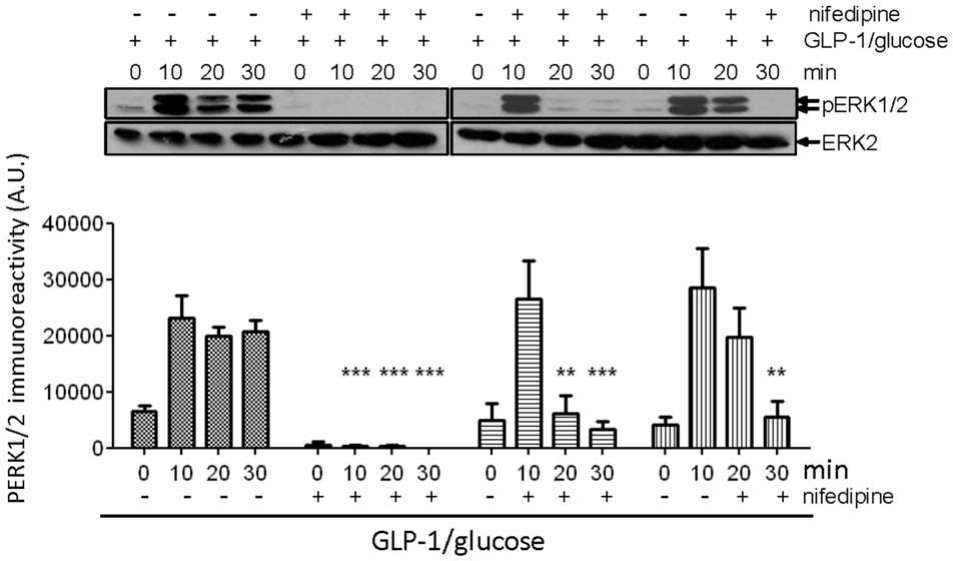
The sustained activation of L-type VGCCs is required for the sustained activation of ERK. MIN6 cells were preincubated for 1 h in KRB supplemented with 1 mM glucose. The cells were then incubated in KRB containing 10 nM GLP-1 and 16.7 mM glucose in the absence or presence of 10 µM nifedipine applied at either 0, 10 or 20 min post-GLP-1/glucose addition. Proteins were resolved by SDS-PAGE and Western blotted using anti-phospho-ERK1/2 (pERK) and anti-ERK2 (ERK2) antibodies. A representative blot is shown with densitometric analysis of the results below showing mean +S.E.M. (n = 3). Results were analysed using two-way ANOVA with Bonferroni's multiple comparison test compared to GLP-1 plus glucose; **, *P*<0.01; ***, *P*<0.001.

To determine whether the sustained activation of L-type VGCCs is required for the sustained activation of ERK, MIN6 cells were treated with GLP-1 plus glucose in the presence or absence of nifedipine for up to 30 min. Nifedipine was applied to the cells at the same time as GLP-1 or 10 and 20 min post-GLP-1 plus glucose treatment and incubated for up to 30 minutes (Figure 2). GLP-1 plus glucose led to an increase in ERK phosphorylation which was inhibited by nifedipine at all times post-nifedipine application (Figure 2). These results demonstrate that the sustained activation of L-type VGCCs is required for the sustained activation of ERK.

### The role of intracellular Ca^2+^ stores in GLP-1-stimulated ERK activation

**Figure 3:**
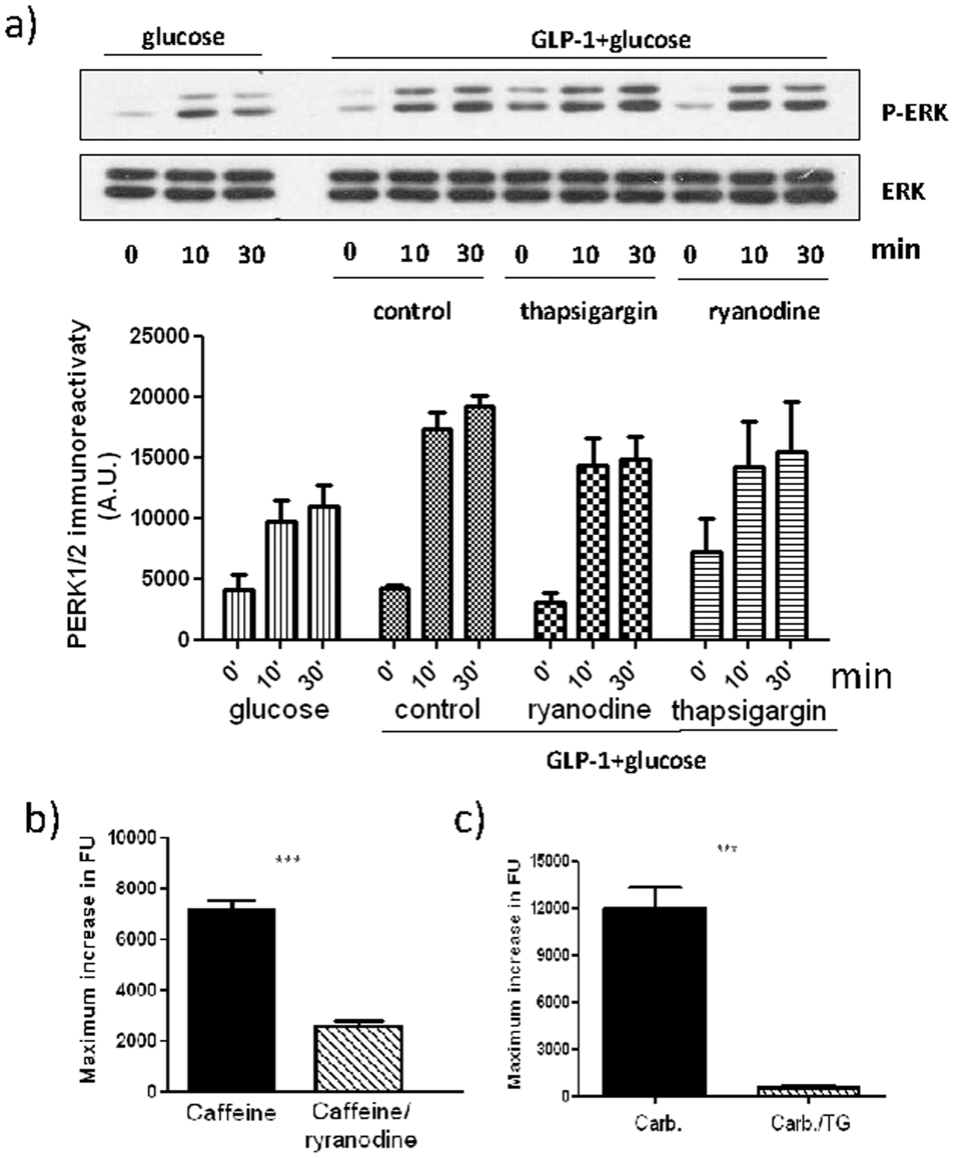
The role of intracellular Ca^2+^ stores in GLP-1-stimulated ERK activation. a) MIN6 cells were either pre-incubated in KRB supplemented with 1 mM glucose in the absence (control) or presence of 100 µM ryanodine or 1 µM thapsigargin for 30 min prior to treatment with 10 nM GLP-1 plus 16.7 mM glucose for the times indicated. Where indicated, cells were also treated with 16.7 mM glucose alone. Proteins were separated by SDS-PAGE and Western blotted using anti-phospho-ERK1/2 (pERK) and anti-ERK1/2 (ERK1/2) antibodies. A representative blot is shown with densitometric analysis of the results below showing mean +S.E.M. (n = 3). Data were analysed by two-way ANOVA with Bonferroni's multiple comparison test compared to GLP-1 plus glucose at each time point. No significant differences were observed. b) MIN6 cells incubated in the absence of extracellular Ca^2+^ calcium were preincubated without or with 100 µM ryanodine for 30 min prior to the addition 10 mM caffeine. Changes in fluorescence as an index of [Ca^2+^]_i_ were determined in fluo-4-loaded cells using a NOVOstar platereader. c) MIN6 cells incubated in the absence of extracellular Ca^2+^ were pretreated without or with 1 µM thapsigargin for 30 min prior to the addition 100 µM carbachol (Carb.) . Changes in [Ca^2+^]_i_ were determined in fluo-4-loaded cells using a NOVOstar platereader ***, *P*<0.001 by Student's t test.

Our data indicate that increases in [Ca^2+^]_i_, mediated by the influx of Ca^2+^ though L-type VGCC are necessary for GLP-1 plus glucose-stimulated ERK activation. However, it is also possible that ERK activation is mediated by Ca^2+^ induced Ca^2+^ release (CICR) from intracellular stores triggered by Ca^2+^ entry through L-type VGCCs. Indeed, it has been reported that glucose plus- GLP-1-stimulated ERK activation in INS-1 cells is dependent upon CICR [3], [14]. To investigate the role of endoplasmic reticulum (ER) Ca^2+^ stores in GLP-1 plus glucose-stimulated ERK activation, MIN6 cells were pre-incubated with ryanodine, to inhibit ryanodine receptors and thus CICR, or thapsigargin to inhibit ER Ca^2+^-ATPase and to cause ER store depletion (Figure 3). The cells were then incubated with 10 nM GLP-1 plus 16.7 mM glucose. However, these inhibitors had no significant effect on the GLP-1 plus glucose-stimulated ERK phosphorylation over the 30 min time-course of the experiment (Figure 3a). The effectiveness of the thapsigargin and ryanodine in blocking IP_3_ and RyR dependent Ca^2+^ efflux respectively from ER-Ca^2+^ stores was confirmed using either carbachol, which robustly stimulates IP_3_-dependent release of Ca^2+^ from intracellular stores via activation of the muscarinic M_3_ receptor [15] (Figure 3c) or caffeine which stimulates Ca^2+^ release via activation of the RyR [16] (Figure 3b). These data provide evidence that Ca^2+^ release from the ER is not essential for L-type VGCC signalling to ERK in MIN6 cells.

### A sustained increase in [Ca^2+^]_i_ is insufficient for the sustained activation of ERK

**Figure 4:**
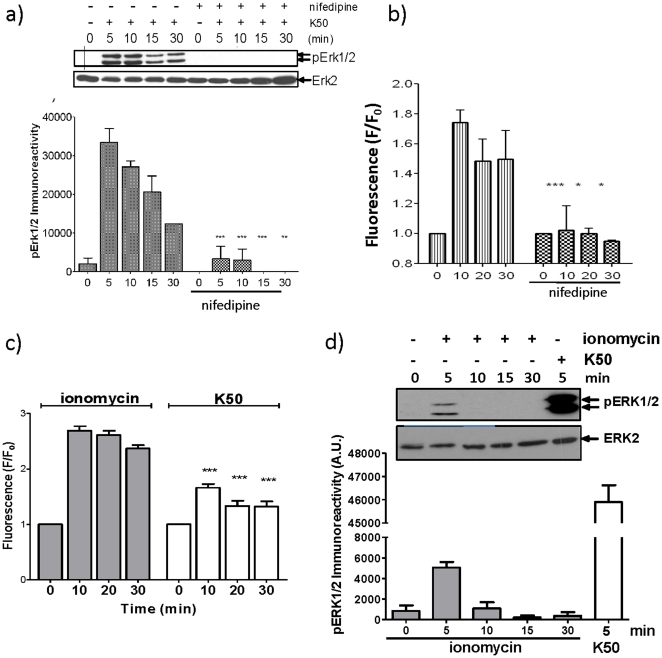
A sustained global increase in [Ca^2+^]_i_ is insufficient for the prolonged activation of ERK. (a and b) MIN6 cells were preincubated for 1 h in KRB supplemented with 1 mM glucose. Cells were incubated in 50 mM K^+^ (K50) in the presence or absence of 10 µM nifedipine for the times indicated (All statistical comparisons were by one-way ANOVA with Bonferroni's multiple comparison test compared to K50 at each time point; *, *P*<0.05; **, *P*<0.01; ***, *P*<0.001). a) Proteins were resolved by SDS-PAGE and Western blotted using anti-phospho-ERK1/2 and anti-ERK2 antibodies. A representative blot is shown above densitometric analysis of the results showing mean +S.E.M. (n = 3). b) In cells loaded with 2 µM fluo-4-AM, fluorescence (as an index of [Ca^2+^]_i_) was measured using a NOVOstar platereader. (c and d) MIN6 cells were preincubated for 1 h in KRB supplemented with 1 mM glucose. Cells were then treated with 10 µM ionomycin or 50 mM K^+^ (K50) for the times indicated. c) In cells loaded with 2 µM fluo-4-AM, fluorescence (as an index of [Ca^2+^]_i_) was measured using a NOVOstar platereader. ***, *P*<0.001 for K50 versus ionomycin at equivalent time points. d) After treatments, proteins were resolved by SDS-PAGE and Western blotted using anti-phospho-ERK1/2 (pERK) or anti-ERK2 (ERK2) antibodies. A representative blot is shown with densitometric analysis of the results below showing mean +S.E.M. (n = 3).

Our data indicate that there is a temporal correlation between an elevation in [Ca^2+^]_i_ via L-type VGCC activation and ERK phosphorylation. Therefore, it is possible that the sustained L-type VGCC-dependent phosphorylation of ERK, observed upon depolarisation, is due to the long-lasting current maintained by this channel and thus its ability to maintain sustained elevations in [Ca^2+^]_i_ (Figure 2). Indeed, an elevation in extracellular potassium results in the robust increase in [Ca^2+^]_i_ and the activation of ERK, which are blocked by nifedipine (figure 4a and b). However, to investigate whether sustained increases in [Ca^2+^]_i_, independent of L-type VGCC activation, are sufficient for sustained ERK activation, we chronically elevated [Ca^2+^]_i_ by treating the cells with ionomycin, a Ca^2+^ ionophore. Although ionomycin caused a sustained elevation of [Ca^2+^]_i_ (Figure 4c), it was unable to induce the sustained activation of ERK (Figure 4d). However, ionomycin was able to cause the transient phosphorylation of ERK (Figure 4d) but the intensity of this phosphorylation was substantially less than that seen in response to elevated extracellular concentration of K^+^ (50 mM), which caused a much lower increase in [Ca^2+^]_i_ through membrane depolarisation and L-type VGCC channel activation (Figure 4). These data provide evidence that L-type VGCC signalling to ERK occurs via a mechanism that is not solely dependent upon the sustained global increase in [Ca^2+^]_i_ but is integrally linked to the activation of L-type VGCCs.

### Local Ca^2+^ influx within the microdomain of the L-type VGCC is sufficient to activate ERK

**Figure 5:**
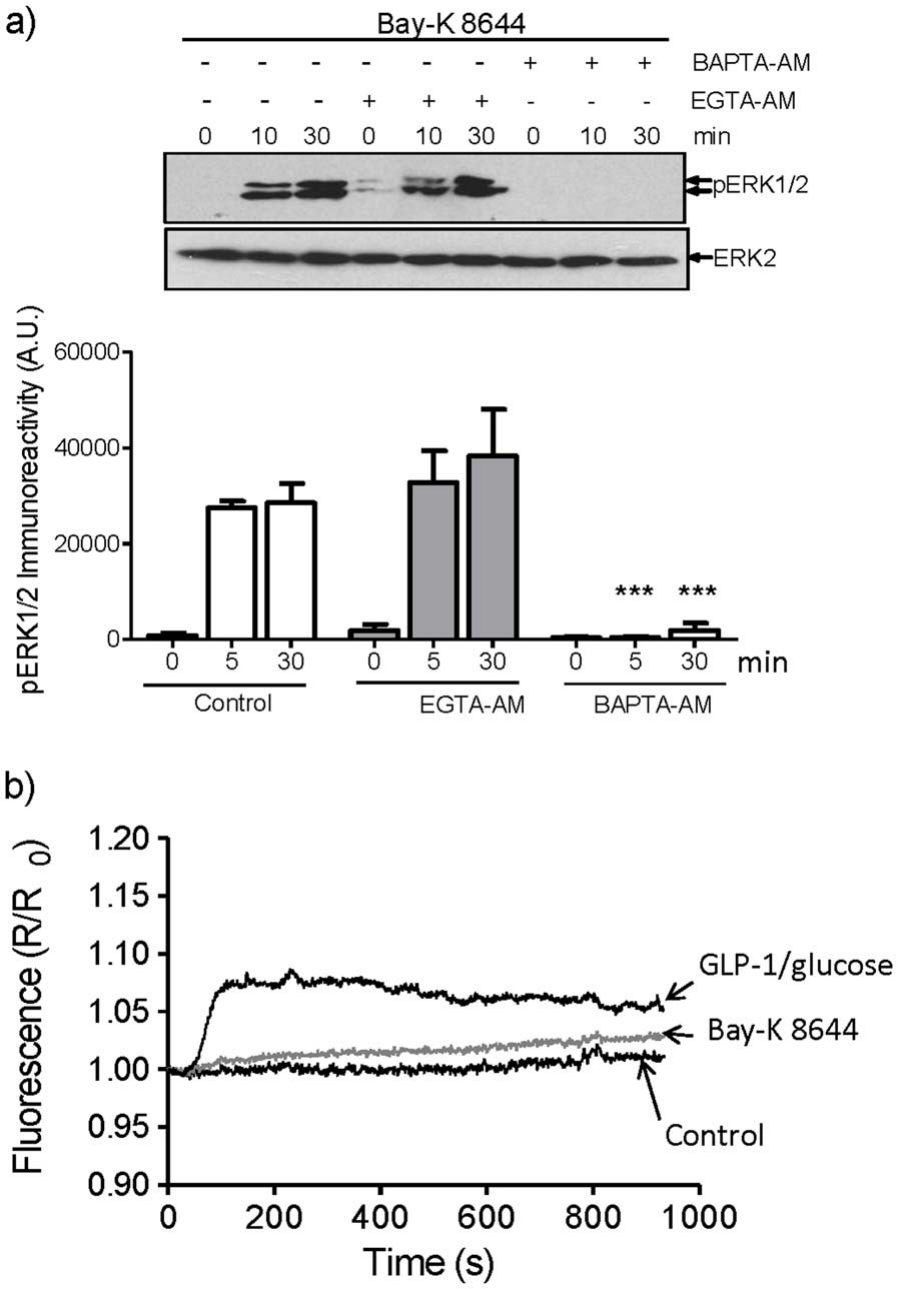
L-type VGCC activation is sufficient to mediate sustained ERK activation in MIN6 cells via local Ca^2+^ signalling. MIN6 cells incubated in KRB plus 2 mM glucose were loaded with 100 µM of EGTA-AM or BAPTA-AM prior to treatment with 10 µM Bay-K 8644 at room temperature for the times indicated. a) Proteins were resolved by SDS-PAGE and Western blotted using anti-phospho-ERK1/2 (pERK) or anti-ERK2 (ERK2) antibodies. A representative blot is shown with densitometric analysis of the results below showing mean +S.E.M. (n = 4). Results were analysed by two-way ANOVA with Bonferroni's multiple comparison test compared to Bay-K 8644 alone at each time point; ***, *P*<0.001. b) MIN6 cells were treated as in (a) but in addition were loaded with 2 µM fura-2-AM and [Ca^2+^]_i_ levels measured by epifluorescence microscopy (n>30). A mean trace is shown. In addition, a representative trace obtained from MIN6 cells stimulated with 10 nM GLP-1 plus 16.7 mM glucose was included for comparison.

To further investigate how L-type VGCCs signal to ERK, MIN6 cells were incubated with Bay-K 8644, an activator of the L-type channel, and changes in the phosphorylation of ERK and [Ca^2+^]_i_ were determined (Figure 5). Bay-K 8644 stimulated the sustained activation of ERK (for up to 30 min) (Figure 5a) yet caused no significant increase in global [Ca^2+^]_i_ (Figure 5b) . The reason Bay-K 8644 does not cause an increase in global [Ca^2+^]_i_ is that under the conditions used in these experiments (i.e. at low glucose concentration) the open probability of the channel is low and therefore Bay-K 8644 only increases the open probability of a small proportion of L-type VGCCs. These results suggests that a local increase in [Ca^2+^]_i_ within the vicinity of the L-type VGCC is sufficient to couple to the ERK signalling pathway. To explore this possibility, MIN6 cells were loaded with BAPTA-AM or EGTA-AM that differ in their rate of Ca^2+^ binding and can reveal information regarding the spatial dynamics of Ca^2+^ signalling (i.e. BAPTA buffers bind and release Ca^2+^ ions approximately 50–400 times faster than EGTA and thus rapidly buffer potential increases in Ca^2+^ at the site of entry) [17]–[21]. When MIN6 cells were treated with Bay-K 8644, the phosphorylation of ERK was unaffected by EGTA-AM but abolished by BAPTA-AM (Figure 5a). This provides evidence that local Ca^2+^, predicted to be within 20 nm of the site of Ca^2+^ entry (i.e. the L-type VGCC) [22], is sufficient to activate ERK in response to Bay-K 8644.

### Local Ca^2+^ influx within the microdomain of the L-type VGCC mediates the sustained GLP-1-stimulated activation of ERK

**Figure 6:**
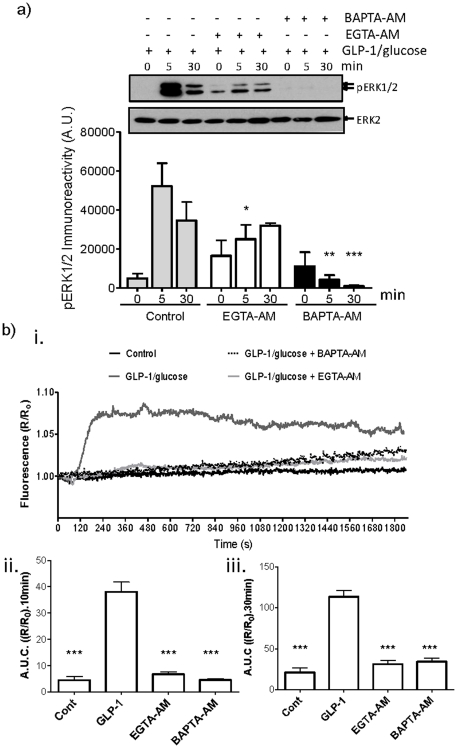
GLP-1-stimulated ERK activation in MIN6 cells is mediated via local Ca^2+^ signalling. MIN6 cells incubated in KRB plus 1 mM glucose were loaded with 100 µM EGTA-AM or BAPTA-AM at room temperature prior to treatment with 10 nM GLP-1 plus 16.7 mM glucose for the times indicated. a) Proteins were resolved by SDS-PAGE and Western blotted using anti-phospho-ERK1/2 (pERK) and anti-ERK2 (ERK2) antibodies. A representative blot is shown with densitometric analysis of the results below showing mean +S.E.M. (n = 3). Data were analysed by two-way ANOVA with Bonferroni's multiple comparison test compared to GLP-1 plus glucose at each time point; *, *P*<0.05; **, *P*<0.01; ***, *P*<0.001. b) MIN6 cells were treated as in a) but in addition, loaded with 2 µM fura-2-AM and [Ca^2+^]_i_ levels measured by epifluorescence microscopy. i) The mean increase in [Ca^2+^]_i_ represented as area under the curve (A.U.C.) during ii) 10 min or iii) 30 min stimulation, mean +S.E.M. (n>30). Statistical comparisons were by one-way ANOVA with Dunnett's range test compared to GLP-1 plus glucose in the absence of chelator. ***, *P*<0.001.

To investigate whether the GLP-1 plus glucose-stimulated ERK activation was also dependent upon local increases in [Ca^2+^], MIN6 cells were incubated with BAPTA-AM or EGTA-AM prior to GLP-1 plus glucose treatment (Figure 6). GLP-1 plus glucose-stimulated increases in global [Ca^2+^]_i_ were effectively buffered by either BAPTA-AM or EGTA-AM (Figure 6b). In addition, GLP-1 plus glucose-stimulated phosphorylation of ERK at 5 and 30 min was completely inhibited by BAPTA-AM, demonstrating that this ERK phosphorylation is dependent upon Ca^2+^. However, in the presence of EGTA-AM, GLP-1 plus glucose-stimulated ERK phosphorylation at 5 min, although significantly inhibited, remained elevated. In contrast, GLP-1 plus glucose-stimulated ERK phosphorylation was unaffected at 30 min (Figure 6a). This indicates that the sustained activation of ERK (30 min) is likely mediated by increased local [Ca^2+^] at the site of entry (i.e. L-type VGCCs) whereas the acute activation of ERK is mediated by both local and global rises in [Ca^2+^].

## Discussion

The present study shows that GLP-1 plus glucose-stimulated ERK activation in pancreatic β-cells is dependent upon the influx of Ca^2+^ through L-type VGCC (Figure 1) and that the sustained activation of the L-type VGCC is critical for the sustained activation of ERK (Figure 2), demonstrating that the two processes are tightly coupled. Although, the sustained activation of L-type VGCCs leads to a sustained rise in the [Ca^2+^]_i_ (Figure 2), global elevations in [Ca^2+^]_i_ alone are insufficient to induce the sustained activation of ERK (Figure 4). Interestingly, pharmacological activation of the L-type VGCC using Bay-K 8644 is able to robustly activate ERK via a Ca^2+^-dependent mechanism but under the non-polarising conditions used in these experiments is unable to stimulate global increases in [Ca^2+^]_i_ (Figure 5). Using BAPTA-AM and EGTA-AM to explore the spatial dynamics of Ca^2+^ signalling to ERK in response to Bay-K 8644, we provide evidence that a local rise in [Ca^2+^] within the microdomain of the L-type VGCC is sufficient to activate ERK (Figure 5). Furthermore, our results show that local increases in [Ca^2+^] within the microdomain of the L-type VGCC are also important in GLP-1 plus glucose signalling to ERK (Figure 6). In this context, both global and local Ca^2+^ signalling to ERK is required for the acute activation of ERK (5 min post-GLP-1 plus glucose stimulation) whereas local increases in [Ca^2+^] are responsible for the prolonged activation of ERK (30 min post-GLP-1 plus glucose stimulation). This indicates that there are two mechanistically distinct phases of ERK activation. Interestingly, two phases of ERK activation in response to GLP-1 plus glucose have been reported previously: a PKA-dependent pathway mediating rapid and transient ERK1/2 phosphorylation and a second β-arrestin 1-dependent phase [23].

In cultured cortical neurones, the L-type VGCC has been implicated in the activation of ERK via a mechanism that requires calmodulin (CaM) binding to the α_1_c (Ca_V_1.2) pore forming subunit of the channel [24]. CaM is thought to bind to a motif on the C-terminal tail of the Ca_v_1.2, the LA motif, within the pore of the α1 subunit in a Ca^2+^-independent fashion [25]. Upon membrane depolarisation and the opening of L-type VGCC, the increase in [Ca^2+^] activates the bound CaM, which is then displaced from the LA motif and re-binds to a second Ca^2+^-CaM binding site (the IQ motif) within the intracellular C-terminal tail of the channel [25]. The bound CaM then either directly activates other signalling molecules and/or causes a conformational change in the channel, which helps it recruit and perhaps activate other signalling molecules [24]. Interestingly, GLP-1 plus glucose-mediated ERK activation in β-cells is also dependent on CaM as W7, a competitive inhibitor of CaM, prevents activation in MIN6 and INS-1E cells [3], [4]. Therefore, the mechanism by which L-type VGCC activation leads to ERK activation in pancreatic β-cells is likely to be similar to that observed in neurones.

There are several downstream effectors of CaM, which may in turn activate ERK indirectly, including the CaM kinases, CaMKII and IV [26] . Indeed, there are a number of reports that show that the CaMKII inhibitors KN62 and KN93 prevent GLP-1 plus glucose-mediated ERK activation in pancreatic β-cells [3], [4]. However, these compounds have been reported to also inhibit voltage-dependent channels [27] and therefore any conclusions reached using these inhibitors should be treated with caution. More recently, calcineurin has been implicated in the activation of ERK in response to glucose in β-cells and intriguingly its activity is also regulated by CaM [28].

Insulin secretion also depends upon a localized increase in [Ca^2+^] close to the VGCCs [29], [30]. The localization of secretory granules close to VGCCs guarantees that granules are exposed to a very high [Ca^2+^], thus ensuring that exocytosis is triggered by the localized increases in [Ca^2+^]_i_ evoked by an action potential. Functional studies using dihydropyridine resistant α_1D_ and α_1C_ channels expressed in pancreatic β-cells indicate that GLP-1 potentiation of glucose-induced insulin secretion is preferentially coupled to the α_1D_ channel in INS-1 cells via a mechanism that requires the activation of ERK1/2 [31].

In conclusion, we show that that the sustained activation of ERK in response to GLP-1 in the presence of an elevated glucose concentration requires the continual activation of the L-type VGCC yet does not require a sustained increase in global [Ca^2+^]_i_ or Ca^2+^ efflux from the ER. Interestingly, based on the studies with BAPTA-AM and EGTA-AM, we provide evidence that elevation of [Ca^2+^]_i_ within 20 nm of the site of entry through L-type VGCCs is sufficient for ERK activation and plays an important role in ERK activation in response to GLP-1.

## Materials and Methods

### Chemicals

Nifedipine, dantrolene, ryanodine and thapsigargin were purchased from Calbiochem (CA, USA). Ionomycin was purchased from Tocris Bioscience (Bristol, UK). EGTA-AM and BAPTA-AM were purchased from Invitrogen (Paisley, UK). All other chemicals (unless stated) were obtained from Sigma-Aldrich (Poole, UK).

### Cell Culture

Mouse Insulinoma-6 cells (MIN6 cells) [12] (kindly provided by Prof. Jun-Ichi Miyazaki) were used between passages 25–40 at ∼80% confluence and grown as described previously [32].

### Cell Treatments

Prior to treatment, the medium was removed and cells washed twice with HEPES-buffered Krebs-Ringer bicarbonate (KRB) (115 mM NaCl, 5 mM KCl, 10 mM NaHCO_3_, 2.5 mM MgCl_2_, 2.5 mM CaCl_2_, 20 mM HEPES, pH 7.4). The cells were then incubated for 1 h at 37°C in KRB containing 1 mM glucose prior to treatments for the times indicated. All treatments were stopped by the addition of ice cold lysis buffer (1% Triton, 10 mM β-glycerophosphate, 50 mM Tris-HCl, pH7.5, 1 mM EDTA, 1 mM EGTA, 1 mM sodium orthovanadate, 1 mM benzamidine HCl, 0.2 mM phenylmethylsulfonyl fluoride, 1 µg/ml each of leupeptin and pepstatin, 0.1% β-mercaptoethanol, and 50 mM sodium fluoride). The lysates were then centrifuged for 10 min at 16,000×*g*, supernatants kept, and total protein concentrations determined by the Bradford assay (Bio-Rad (CA, USA)). The protein lysates were stored at 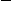
80°C until further analysis.

### SDS-Polyacrylamide Gel Electrophoresis (PAGE) and Immunoblotting

SDS-PAGE and Western blotting were performed as previously described [32]. Rabbit anti-phospho-ERK1/2 antibody (recognizing only the phosphorylated (activated) forms of ERK) and anti-ERK2 antibody were purchased from New England Biolabs (Hitchin, UK). Detection was by horseradish peroxidase-linked anti-rabbit secondary antibodies and enhanced chemiluminescence (GE healthcare, (Bucks, UK)).

### Single Cell Ca^2+^ Imaging

For Ca^2+^ imaging, MIN6 cells were loaded with 2 µM fura-2-acetoxymethyl ester (fura-2-AM), prepared in dye-loading buffer (KRB supplemented with 1 mg/ml bovine serum albumin (BSA) and 0.1% Pluronic F-127) for 30 min at 20°C. The cells were then washed and further incubated for 10 min in KRB to allow de-esterification of the indicator. Changes in fluorescence were monitored using a Nikon Diaphot 200 inverted epifluorescence microscope with an oil immersion objective (40×) and a SpectraMASTER II module (PerkinElmer Life and Analytical Sciences, Waltham, MA). Cells were excited alternatively with light at 340 nm and 380 nm with emission detected at 510 nm. Raw fluorescence data were exported to Microsoft Excel and expressed as the 340/380 ratio for each cell and further analysed in GraphPad Prism. A ratio value was collected each 2 s. Data are reported as either the 340/380 ratio (R) or R/R_0_ where R_0_ is the basal (unstimulated) ratio.

### Population NOVOstar [Ca^2+^]_i_ measurement

Population-based Ca^2+^ fluorescence measurements were performed using a NOVOstar microplate reader (BMG LabTechnologies, Offenburg, Germany) equipped with a pipettor and 2 injectors. MIN6 cells in a 96-well plate format were washed twice with KRB buffer then loaded with 2 µM fluo-4-AM as described above. Cells were washed with KRB after fluo-4-AM loading. Details of treatments are provided in the figure legends. Fluo-4 was excited using a high energy xenon flash lamp through a 485 nm filter and emission detected by a photomultiplier after passing through a 520 nm filter. Measurements were collected at 2 s intervals. Raw fluorescence data were exported to Microsoft Excel and expressed as *F*/*F*_o_ (stimulated fluorescence/basal fluorescence) and further analysed in GraphPad Prism. A single well of cells without fluo-4-AM loading was used as an auto-fluorescence control and subtracted from the fluorescence values obtained. Data are reported as the average of at least three independent experiments ± S.E.M..

### Electrophysiology

MIN6 cells were preincubated for 1 h in KRB buffer prior to electrophysiological measurements. Membrane potential was recorded using an Axopatch 200 amplifier (Axon Instruments (CA, USA)). Signals were digitized using a DIGIDATA 1322A interface, and records acquired and analysed using pClamp9.2 software (Axon Instruments (CA, USA)). All electrophysiological recordings were made at 28°C using the perforated-patch, whole-cell configuration. Patch pipettes (3–5 MΩ) were made from thick-walled borosilicate glass. Pipettes were initially dipped in a solution containing: 76 mM K_2_SO_4_, 10 mM NaCl, 10 mM KCl, 1 mM MgCl_2_, and 5 mM HEPES (pH 7.35 with KOH) and then back-filled with the same solution including amphotericin B (240 µg/ml). The bath solution contained: 140 mM NaCl, 3.6 mM KCl, 2 mM NaHCO_3_, 0.5 mM NaH_2_PO_4_, 0.5 mM MgSO_4_, 2.6 mM CaCl_2_, 5 mM HEPES and 2.5 mM D-glucose (pH 7.4 with NaOH).

### Statistical Analysis

Statistical differences between multiple groups were analysed by either one-way or two-way analysis of variance (ANOVA), followed by post-hoc analysis. Comparison between two sets of data have been analysed by Student's t-test. Statistical analyses were performed in GraphPad Prism and significance was only confirmed when p<0.05.
